# The Impact of Transient Ischemic Attack (TIA) on Brain and Behavior

**DOI:** 10.3389/fnbeh.2019.00044

**Published:** 2019-03-11

**Authors:** Leif E. R. Simmatis, Stephen H. Scott, Albert Y. Jin

**Affiliations:** ^1^Centre for Neuroscience Studies, Queen’s University, Kingston, ON, Canada; ^2^Department of Medicine, Queen’s University, Kingston, ON, Canada; ^3^Department of Biomedical and Molecular Sciences, Queen’s University, Kingston, ON, Canada

**Keywords:** transient ischemic attack (TIA), cognition, behavior, MRI, EEG, functional connectivity, robotics

## Abstract

Transient ischemic attack (TIA) was originally defined as self-resolving focal cerebral ischemia with symptoms lasting <24 h. The newer definition also added the limitation that there should be no evidence of acute brain tissue infarction, to recognize that acute injury to the brain can result from ischemia of <24-h duration. However, several recent findings suggest that having a TIA correlates with deficits that can persist far beyond the resolution of clinical symptoms, even in the absence of imaging evidence of ischemic tissue injury. These deficits may be the result of subtle perturbations to brain structure and/or function that are not easily appreciated using the standard clinical and imaging tools that are currently employed in practice. Here, we will discuss evidence that suggests that TIA may lead to lasting changes to the structure and function of the brain.

## Introduction

Currently it can be argued that the approach to investigating transient ischemic attack (TIA) and minor stroke is a compromise between timeliness of investigation and accuracy of diagnosis. Diagnostic accuracy hinges on identification of tissue injury, i.e., infarction. However, the tools that can be used to achieve this degree of accuracy, e.g., magnetic resonance imaging (MRI), are not always readily available at the point of care. This dilemma is also linked to the evolving definition of TIA and stroke, which has changed over the past few decades to accommodate both clinical utility and biological significance. The initial description of TIA as a transient neurological deficit of suspected vascular origin lasting <24 h has been used widely throughout epidemiological studies and for pragmatic clinical decision-making. As such, it has been a useful construct that could be applied in any clinical setting requiring nothing more than the clinician’s judgment from the patient’s history and examination findings, facilitating rapid diagnosis. However, this definition has been abandoned in light of recent imaging data that demonstrates that about 50% of these patients have evidence of infarction on diffusion-weighted MRI (DWI; Easton et al., 2009). Therefore, TIA has been re-defined as a transient neurological deficit of any duration without evidence of parenchymal injury, while symptoms that are accompanied by evidence of infarction now constitute stroke and portend a much greater risk of recurrent stroke (Easton et al., 2009). While the new definitions of TIA and stroke are much more accurate regarding the biological consequences of cerebral ischemia (Johnston et al., 2000; Kleindorfer et al., 2005; Coutts et al., 2012), they create new dilemmas for clinicians and investigators. The new TIA definition is heavily reliant on MRI scan, which is not readily available in most Emergency Departments on a 24-h basis, making it more difficult to differentiate TIA from stroke. Conversely, patients without parenchymal damage often have uneventful recoveries and many are likely to not have had cerebral ischemia at all but rather a mimic of stroke or TIA, such as migraine (Prabhakaran et al., 2008). In these patients, the diagnostic label of TIA is therefore inaccurate and could lead to unnecessary investigations and interventions. Despite recent remarkable advances in imaging cerebral ischemia, the discordance between clinical utility (i.e., rapid diagnosis) and biological insight (i.e., accurate assessment of tissue injury) is not fully resolved. In 2013, the American Heart Association (AHA) proposed a modification of the TIA definition to allow for the diagnosis of TIA to be made without MRI (Sacco et al., 2013) but this still does not clarify the possibility of concurrent parenchymal damage, as computed tomography (CT) alone is insufficient to rule this out. For those who have no evidence of infarction on MRI scan, it is often unclear if these patients had transient cerebral ischemia at all. Further confounding these issues is recent evidence showing that some TIA patients may experience persistent deficits undetected by conventional clinical and imaging assessments. Markers of brain injury following TIA remain an active area of research but to date no supplementary tests have been approved for use in clinical practice.

Current techniques that are used in the diagnosis of stroke and TIA have important limitations for both rapid clinical decision-making and for understanding the functional consequences of cerebral ischemia. In this review article, we will examine the role that anatomical imaging, neurophysiological investigations and functional/behavioral assessment may have in providing accurate biological insight in a timely manner for TIA/minor stroke diagnosis.

## Current Brain Imaging Techniques in the Assessment of TIA

CT was the first tool to identify acute ischemic structural injury to the brain after transient symptoms lasting <24 h (Perrone et al., 1979). It was later shown that the presence of acute ischemic CT lesions correlated with worse functional outcomes and longer symptom duration, even when symptoms resolved in <24 h (Coutts et al., 2012), and that CT lesions indicate a greater risk of stroke within 90 days (Wasserman et al., 2015). Thus, individuals experiencing transient symptoms lasting <24 h could still experience permanent damage to the brain and are at greater risk of future cerebrovascular events. CT remains the most widely used clinical imaging tool for identifying those in need of immediate care, i.e., those who have acute ischemic infarction, because of its speed of administration and ease of access in modern Emergency Departments. The primary drawbacks to CT are its relatively low sensitivity for detecting minor acute ischemic lesions (Lansberg et al., 2000) and the delivery of substantial doses of ionizing radiation with each scan (Smith-Bindman et al., 2009).

MRI and DWI have become the methods of choice in TIA and minor stroke diagnosis; however, DWI has key limitations that make it problematic to rely upon it as a sole diagnostic tool. DWI has better sensitivity than CT for identifying ischemic injury to the brain (Mintorovitch et al., 1991; Kidwell et al., 1999; Sorensen and Ay, 2011; Moreau et al., 2013) and it can detect relevant ischemic lesions in approximately 50% of individuals who have transient neurological symptoms lasting <24 h (Coutts et al., 2005; Moreau et al., 2013), many of whom are CT-negative. Therefore, many of the individuals who would have received a diagnosis of “TIA” based on CT findings actually had strokes based on DWI findings. Indeed, a DWI lesion is likely to be found in an individual that has symptoms lasting >1 h (Crisostomo et al., 2003; Calvet et al., 2009) and indicates an elevated risk of subsequent stroke (Rothwell et al., 2005). These findings led to a redefinition of TIA in 2009 by the AHA (Easton et al., 2009) with greater emphasis on tissue injury and recommendation of DWI for diagnosis. Unfortunately, up to two-thirds of individuals who are acutely DWI-positive will no longer show evidence of infarction at 21 days (Schulz et al., 2003). The DWI signal fades quickly reducing the sensitivity of DWI within 9 days. Endogenous tissue recovery processes such as oedema reduction are likely the cause of this (Katzman et al., 1977). This means that delayed imaging in an individual who would have been diagnosed with stroke could instead lead to an incorrect diagnosis of TIA. Also, up to a quarter of individuals with lesion-free TIA show increases in their modified Rankin Scale (mRS) score 90 days after their index event (Coutts et al., 2012). Thus, while the imaging criteria for TIA may be fulfilled with a DWI-negative scan, this does not mean that a given patient will be functionally normal after their index event. Lastly, there remains a false-negative rate of about 7% for DWI in acute stroke (Edlow et al., 2017).

Some of the technical shortcomings of DWI can be overcome with the use of MRI sequences such as fluid-attenuated inversion recovery (FLAIR). In some cases, FLAIR will be positive for a clinically-relevant lesion after 30 days even though DWI was negative <24 h after symptom onset (Sylaja et al., 2008), thus prolonging the window of lesion detection. Additionally, up to 70% of DWI lesions can be found on FLAIR 10 months after symptom resolution (Oppenheim et al., 2006), indicating permanent injury that is not obvious on DWI. FLAIR in conjunction with DWI also allows lesions to be identified that would not be obvious on DWI alone (Oppenheim et al., 2000; Lansberg et al., 2001). FLAIR therefore is a promising tool for investigating structural injury after TIA and captures some markers of brain tissue injury that are missed when using DWI alone. Unfortunately, FLAIR suffers from the same equipment-availability limitations as all other MRI-based tools.

More-advanced 3-dimensional MRI approaches such as diffusion tensor imaging (DTI) can now identify injury to white matter (WM) tracts (Le Bihan et al., 2001) in people who have had transient symptoms and yet do not show injury on DWI or other conventional MRI sequences. Although the diagnostic value of these techniques remains unproven, these approaches can theoretically improve detection of abnormalities in people diagnosed with TIA. For example, decreased fractional anisotropy (relative to healthy controls) in the anterior thalamic radiations has been found in people diagnosed with transient sensorimotor symptoms despite an absence of DWI lesions 90 days after symptom resolution (Ferris et al., 2017). Frontal WM damage has been shown to correlate with performance on the Montreal Cognitive Assessment (MoCA) and Mini-Mental State Exam (MMSE) in people who had lesion-free transient neurological symptoms (Zamboni et al., 2017). Thus, tools to investigate structural connectivity offer promise for identifying abnormalities in people diagnosed with TIA, although practical limitations such as availability and the prognostic value of these techniques with respect to future risk of stroke and functional decline remain unresolved.

## Disruption in Brain Network Functionality

Disruption of the normal function of the brain is becoming a recognized effect of transient cerebral ischemia and relates to behavioral outcomes (Coutts et al., 2012; van Rooij et al., 2014). While the presence of a new ischemic lesion on imaging remains one of the strongest predictors of subsequent stroke and functional decline (Coutts et al., 2012), being lesion-free does not mean that an individual’s brain has been spared injury; abnormalities may simply be beyond the scope of standard clinical tools or static imaging techniques. In stroke patients, there is evidence for impacted memory and language production even though the affected networks can be remote from lesion sites (Geranmayeh et al., 2016; Siegel et al., 2016). Evaluating network- and behavior-level brain function in TIA in a similar manner may provide more valuable information regarding behavioral domains than static tissue damage, including individuals without obvious DWI lesions. Some of these techniques, or related ones, may eventually augment DWI in a diagnostic sense, although more research is required in this area.

Functional MRI (fMRI) is a valuable tool for assessing changes in brain metabolism or connectivity related to tasks or at rest. In people diagnosed with TIA without T2- or FLAIR (3.0-Tesla) lesions, fMRI has shown reduced resting state network connectivity and reduced dorsolateral prefrontal cortex regional homogeneity for 1 month after symptom resolution (Li et al., 2013; Guo et al., 2014). These abnormalities would not be possible to find using static imaging, yet they point to perturbed brain function in lesion-free individuals diagnosed with TIA. Unfortunately, fMRI results are susceptible to participants’ movements (Van Dijk et al., 2012) which limits the repertoire of testable behaviors. Brain activity can be also be quantified by measuring electrical activity using electroencephalography (EEG) and magnetoencephalography (MEG). An EEG study recently showed that people diagnosed with TIA, most without DWI lesions, had increased focal slow wave activity that persisted for 1 month in some individuals (Bentes et al., 2017), indicating pathology in the corresponding tissue (Britton et al., 2016). This has been corroborated by an MEG study that demonstrated increases in both slow wave (2–6 Hz) and beta (12.5–30 Hz) activity over the affected sensory- and motor cortices in people who had lesion-free TIAs with sensorimotor symptoms (Stippich et al., 2000). MEG and EEG provide information complementary to each other, although they are not fully independent (Malmivuo, 2012). Thus, MEG has a niche role as a tool for investigating network dysfunction but currently requires costly and bulky equipment. Further research using MEG for neurological dysfunction may be facilitated by using compact semi-portable devices that do not require extreme cooling (Boto et al., 2018), however this technology is still far from clinical application. These novel results are promising, but further research is required to evaluate the relationship between brain network functionality, behavior, and long-term functional outcomes in people diagnosed with TIA.

Brain function can be tested in the context of basic muscle control using transcranial magnetic stimulation (TMS). Three common TMS metrics are utilized: cortical silent period (SP), EMG suppression after a single high-intensity TMS pulse; intracortical inhibition (ICI), motor-evoked potential reduction elicited by a low-intensity TMS pulse if it is followed by a higher-intensity one; and intracortical facilitation (ICF), motor-evoked potential increase elicited by high-intensity TMS pulse if it is followed by a lower-intensity one. People diagnosed with TIA have demonstrated both increased ICF and reduced ICI in the affected hemisphere compared to the unaffected hemisphere, without evidence of structural MRI lesions (Koerner and Meinck, 2004; Edwards et al., 2011). SP in these individuals can also be prolonged for those that experience symptoms ≥1 h, in people with or without DWI lesions (Koerner and Meinck, 2004; Wong et al., 2004). These findings suggest that TIA is associated with altered cortical excitability, which may persist despite the absence of overt structural injury to the brain. However, the significance of TMS abnormalities in terms of patient outcomes requires much more research, particularly in individuals without DWI lesions.

## Clinical and Behavioral Measures of Impairment in TIA

Further clues that impairments after TIA can persist long after symptom resolution come from the use of traditional clinical assessments, such as bedside cognitive screening tests. Cognitive deficits on the MMSE or the MoCA can persist for months after lesion-free (CT) TIA (Pendlebury et al., 2011, 2012; van Rooij et al., 2014). Individuals who had lesion-free TIAs may also have an increased chance of experiencing fatigue (Winward et al., 2009). Furthermore, up to 26% of DWI-negative TIA patients have functional decline on the mRS after 90 days (Coutts et al., 2012). While valuable for identifying acute tissue infarction, DWI may be insufficient on its own to predict functional decline. One study found that DWI-negative and DWI-positive individuals were equally likely to have an mRS ≥2 after their event and equally likely to have cognitive impairments after 1 year (Makin et al., 2015). However, while traditional clinical measures highlight that there can be problems after a TIA or minor stroke and can provide additional information over and above a DWI scan, they are coarse and lack sensitivity. For example, the mRS is scored from 0 to 6, with 0 indicating normal function and 6 indicating death. Additionally, much of the existing data for persisting deficits after TIA/minor stroke come from cognitive testing only. Characterizing persisting deficits after TIA or minor stroke in greater detail and in a broader set of domains may help us to better understand the factors that contribute to functional decline in some individuals. Presently it is known that underlying risk factors such as diabetes mellitus, ongoing symptoms, CT/CT angiography positivity, and female sex, could contribute to an increased risk of disability after a TIA (Coutts et al., 2012).

The problem of imprecision in tradition clinical examinations could be addressed with the use of novel technologies. Robotics provide a means by which to investigate behaviors in a more sensitive way than is possible with standard clinical tools and allows the behavioral correlates of neurological injury to be investigated. Recently, a cohort of people who had a first-ever TIA was assessed using the KINARM robot (Simmatis et al., 2017). Symptom durations for all participants were <24 h. The subset of the cohort that was scanned using DWI had no evidence of lesions, although robotic task performances in the scanned group were statistically indistinguishable from those in the un-scanned group. Common impairments across the cohort included cognitive override and the ability to generate quick and accurate voluntary reaching movements. Importantly, this study indicated that persisting deficits after TIA are not limited to cognition. Instead, they can span multiple domains and include motor behaviors, which was previously unrecognized as a problem after TIA. Another recent study identified that gait may be abnormal in people who have TIA or minor stroke for up to 5 months after the index event (Batchelor et al., 2015). However, this study did not differentiate clearly between TIA and minor stroke, which would need to be addressed in future studies of gait abnormalities in people who have had transient cerebral ischemia. Therefore, the spectrum of deficits that are present after a TIA or minor non-disabling stroke may be broader than was thought possible when TIA was first defined decades ago. The identification of subtle behavioral deficits after TIA paves the way for identifying how predictive these are of functional decline. However, the combined diversity and subtlety of persisting symptoms after TIA or minor stroke creates a problem for differentiating those who are at risk of decline from those that will have an uneventful recovery. Perhaps the use of machine learning tools such as deep learning (LeCun et al., 2015) will provide some of these insights. Deep neural networks are starting to be used with some success to identify individuals at risk of tissue injury after stroke and to predict treatment effects (Nielsen et al., 2018). Potentially, the combination of quantitative assessment techniques with advanced statistical/machine learning approaches may help us move beyond the use of DWI as the exclusive diagnostic tool in TIA and minor stroke.

## Conclusions

The current approach to TIA and minor stroke investigation is focused appropriately on immediate threats to health such as recurrent stroke. However, these approaches offer limited insight on how the consequences of transient cerebral ischemia evolve for the patient. Additionally, the ability to identify tissue injury rapidly remains incomplete. The original definition of symptom resolution in <24 h did not account for structural injury to the brain. DWI has vastly improved our capacity to characterize TIA and minor stroke pathophysiology; however, limitations in availability and sensitivity of this and other MRI-based tools should drive the adoption of supplementary diagnostic techniques. Research on tools such as MEG, EEG, robotics, and various MRI-based tools to study brain function after TIA and minor stroke is still a “work in progress,” and presently has no prognostic value. However, with further refinement, these tools could be eventually used to supplement DWI and provide additional diagnostic/prognostic insight. Importantly, there is increasing recognition that there are other consequences to TIA and minor stroke beyond the presence or absence of DWI lesions. A more comprehensive approach incorporating clinical history, imaging, tests of brain function, and associated behavioral consequences after cerebral ischemia could help in the timely and accurate identification of individuals who are likely to suffer recurrent stroke or functional decline.

## Author Contributions

LS was the primary researcher and writer of this manuscript. SS and AJ assisted with writing and editing the manuscript.

## Conflict of Interest Statement

SS is the co-founder and Chief Scientific Officer of BKIN Technologies Ltd., the company that commercializes the KINARM robot that is discussed in this article. The remaining authors declare that the research was conducted in the absence of any commercial or financial relationships that could be construed as a potential conflict of interest.
